# Prevalence and Risk Factors for Myopia in Primary School Children in Madrid: A School-Based Cycloplegic Refraction Study

**DOI:** 10.3390/ijerph22121766

**Published:** 2025-11-21

**Authors:** Maria Nieves-Moreno, Gonzalo Carracedo-Rodriguez, David Pablo Piñero-Llorens, Laura Batres Valderas, Sergio Recalde-Maestre, Javier García-da-Silva, Blanca Díaz-Vega, Sara Llorente-Gonzalez, Maria Alarcón-Tomás, Monica Lovera-Rivas, Sara Gutierrez-Jorrin, Paulina Dotor-Goytia, Patricia Fernández-Robredo, Pilar Gómez de Liaño, Susana Noval-Martin, Macarena Dosal-Franco

**Affiliations:** 1 Department of Pediatric Ophthalmology, IdiPaz, La Paz Universitary Hospital, 28046 Madrid, Spain; maria.nieves@salud.madrid.org (M.N.-M.); susana.noval@salud.madrid.org (S.N.-M.); 2 Ocupharm Research Group, Universidad Complutense de Madrid, 28037 Madrid, Spain; jgcarrac@ucm.es; 3 Department of Optics, Pharmacology and Anatomy, University of Alicante, 03690 Alicante, Spain; david.pinyero@gcloud.ua.es; 4 Department of Optometry and Vision, Faculty of Optics and Optometry, Complutense University of Madrid, 28037 Madrid, Spain; lbatres@ucm.es; 5 Department of Ophthalmology, Clinic of Navarra University, 31008 Pamplona, Spain; srecalde@unav.es (S.R.-M.); sllorente@unav.es (S.L.-G.); 6 Retinal Pathologies and New Therapies Group, Experimental Ophthalmology Laboratory, Department of Ophthalmology, Universidad de Navarra, 31008 Pamplona, Spain; pfrobredo@unav.es; 7 Association of Magna Myopia with Retinopathies—AMIRES, 28002 Madrid, Spain; amires@miopiamagna.org; 8 Gran Visión Oftalmología, 28026 Madrid, Spain; blancadiazvega@yahoo.es; 9 Puerta de Hierro Universitary Hospital, 28222 Madrid, Spain; draalarcontomas@gmail.com; 10 Avalens, 28009 Madrid, Spain; lililovera@hotmail.com; 11 OCULUS Iberia SL, 28760 Madrid, Spain; saragutierrez@oculus.es; 12 Indizen Optical Technologies S.L., 28002 Madrid, Spain; paulina.dotor@gmail.com; 13 General Universitary Hospital Gregorio Marañón, 28007 Madrid, Spain; pilargomezgl@gmail.com

**Keywords:** myopia, high myopia, refraction, cycloplegic refraction, myopia prevalence

## Abstract

Objective: We assess the prevalence of myopia and its associated factors among schoolchildren in Madrid, Spain, where school-based data using cycloplegic refraction are currently scarce. Methods: A cross-sectional study was conducted in 39 randomly selected schools in Madrid, targeting children in the second (6–7 years) and sixth grades (11–12 years). Parents completed questionnaires detailing family ocular history, the child’s lifestyle, and screen time. Socio-economic status was inferred from the Human Development Index of school districts. Children were examined using a two-stage approach: initial screening with visual acuity testing and Plusoptix photorefraction (myopia screening cut-off ≤ 0.00 D spherical equivalent), followed by confirmatory cycloplegic autorefraction (three drops of cyclopentolate 1% administered at 10 min intervals), with myopia defined as spherical equivalent ≤ −0.50 D. Statistical analyses included chi-square tests and logistic regression models to evaluate associated factors. Results: Of 3680 children invited, 2489 (67.6%) were examined. Myopia prevalence was 6.5% in second grade and 18.7% in sixth grade. Family history of myopia was a significant risk factor (OR 2.04; 95% CI: 1.53–2.70; *p* < 0.001 for both parents). Outdoor activity during weekends was associated with lower myopia prevalence (OR 0.50; 95% CI: 0.37–0.66; *p* < 0.01 for 2–6 h). Screen time was not a significant factor in multivariate analysis. Conclusions: This large school-based study using cycloplegic refraction provides more accurate prevalence data for Spanish schoolchildren. It confirms family history as a major risk factor and highlights the association of outdoor activities with lower prevalence of myopia. These results underline the need for preventive measures and suggest areas for future interventional research.

## 1. Introduction

Myopia is becoming an emerging epidemic, with nearly half of the world population projected to be myopic by 2050 [1]. Although in most cases this condition can be corrected with glasses, contact lenses or refractive surgery, severe forms of myopia increase the risk of sight-threatening complications including retinal detachment, strabismus, glaucoma, and macular degeneration, constituting a major public health and socio-economic problem [2].

The prevalence of myopia has geographical, ethnic, and generational differences, with the maximum prevalence in East Asia and the lowest in Africa [3,4]. In China, 80% of high school students have myopia and the prevalence among primary school students increased from 22.53% in 1989–2014 to 38.92% in 2018–2020 using non-cycloplegic refraction [5,6]. Alvarez-Peregrina et al. [7] reported an increase in myopia in children in Spain from 16.8% in 2016 to 19.1% in 2017 using non-cycloplegic refraction. The most up-to-date and comprehensive estimate of the prevalence of myopia in Europe based specifically on cycloplegic refraction is 18.9% (95% CI: 13.2–26.5%) [8]. Globally, the development of myopia in children is associated with hereditary factors, environmental pollution, lifestyle choices, living conditions, less time spent outdoors, and greater use of electronic devices [9,10,11].

Although several studies have shown a significant increase in the prevalence of myopia worldwide, there is a relative paucity of research in this area in Spain. It is essential to continue with epidemiological studies in different populations in order to add information and clarify the prevalence of myopia and high myopia in Europe. Preventive measures against myopia are primarily applicable in childhood and it is also in this stage that myopia progresses most rapidly. Therefore, the aim of this study was to analyze myopia prevalence following cycloplegic refraction in children in second and sixth grades of primary school (aged 6–7 and 11–12) in the city of Madrid and its association with such risk factors as family history, outdoor activities, and screen time.

## 2. Methods and Materials

An epidemiological cross-sectional study was designed and data collection conducted in 39 schools in the city of Madrid. The study adhered to the principles of the Declaration of Helsinki, and the protocol was approved by the Ethics Committee of the Clínica Universidad de Navarra. Children in the second (aged 6–7) and sixth grades (aged 11–12) were selected. Children in second and sixth grades were selected to obtain a large, age-stratified sample within primary education, targeting one group from the early years and another from the final years of this educational stage. First grade was intentionally avoided, as children at that age are still developing social and cooperative skills, which can make participation in structured vision assessments more challenging.

### 2.1. Sample Size

To determine the sample size, an estimated prevalence of high myopia (>6 diopters) of 2% was considered, with a sampling error of ±1 and a 95% confidence level. Additionally, mild to moderate myopia (0.5–6 diopters) was estimated at 10%, with a sampling error of ±2 at the same confidence level [1]. These estimates were based on preliminary data. Considering a design effect of 1.25, the optimal sample size was determined to be 1200 cases per age group, specifically students in 2nd and 6th grade, resulting in a total of 2400 participants. An average of 17 parental consents per classroom was expected, considering an average class size of 25 students and a valid response rate below 70%. Based on this estimate, approximately 140 classrooms will be required, selected from 44 schools, some with a single classroom per grade and others with at least two classrooms for 2nd and two for 6th grade.

### 2.2. Data Collection Process

A total of 44 schools were randomly selected from the total number of schools in the city of Madrid. Of these, 5 schools declined to participate in the study. The data collection process in schools followed these steps:

1. Initial Contact: A formal introductory letter was sent by mail to the principals of the selected schools, explaining the study’s objectives and the level of involvement required from the institution.
2. Phone Confirmation: An initial phone call was made to confirm each school’s participation, verify the number of enrolled students in the selected grades and schedule the first on-site visit.
3. School Visits: A minimum of two to three visits will be conducted at each school:
  - First Visit: A list of participating classrooms was requested to distribute individualized information to the families, including an explanation letter, a parental consent form for the child’s participation, and a brief questionnaire on visual health history and habits.
  - Second Visit (3–5 days later): Received consent forms were be reviewed. Given the expected response rates, a second round of information with the same documents was distributed to students whose parents had not yet responded, aiming to maximize participation.
  - Third Visit: Qualified professionals conducted refractive measurements on children with confirmed parental consent. From each classroom all children whose parents gave consent were included, with no exclusion criteria.

### 2.3. Study Development

A non-validated questionnaire created by a committee of experts (DP, SRC and PGL) was completed by parents days before the examination including questions about family ocular history (myopia and high myopia in the parents), the personal history of the child (previous refractive correction and amblyopia), and lifestyle (number of hours outdoors/indoors and screen time). Parents were asked to respond to the following questions: (1) Number of hours spent on outdoor activities on weekdays and weekends (e.g., soccer, in parks); (2) Number of hours spent on indoor activities that do not require excessive near vision on weekdays and weekends (e.g., basketball, swimming, dance); (3) Number of hours spent on indoor activities that require near vision on weekdays and weekends (e.g., painting, learning a new language, studying) and (4) Number of hours using electronic devices at near distance on weekends and weekdays.

The lifestyle and family history questionnaire was specifically designed for this project and has not been validated. Parents completed the questionnaire independently at home after receiving standardized written instructions. Responses were collected in a categorical format (e.g., <1 h, 1–2 h, >2 h per day), not as free text. No pilot testing phase was conducted prior to implementation. A copy of the questionnaire is provided as Supplementary Material (File S1).

Participants were divided according to their socio-economic status based on the Human Development Index (HDI) of districts in which the schools were located. The HDI published and developed by the Madrid City Council is based on indicators such as income level, level of education, and life expectancy. This stratification is based on four strata corresponding to levels of development, from highest to lowest (upper class, upper middle class, middle class, and lower class). Individual-level socioeconomic data were not collected.

The study was approved by the Navarra University Clinic Ethics Committee. All parents signed the consent form for their children to participate.

During initial screening, children were examined with the Plusoptix photorefractometer (Plusoptix, Plusoptix GmbH, Nuremberg, Germany) at a distance of 1 m. A spherical equivalent (SE) ≤ 0.00 D was considered a positive screening result for possible myopia, and in those children cycloplegic refraction was then performed to avoid errors due to accommodation since Plusoptix tends to overestimate myopia [12]. This second analysis was conducted 45 min after instilling three drops of cyclopentolate hydrochloride 1% (minims, Bausch & Lomb) at 10 min intervals. The efficacy of cycloplegia was confirmed by pupil dilatation and absence of light reflex previously to measure cycloplegic refraction. This was followed by an ophthalmological examination by ophthalmoscopy and measurement of the refraction with an autorefractometer (Myopia Master, OCULOS Iberia S.L., Tres Cantos, Madrid), performed by an optometrist and an ophthalmologist. Subjective refraction was not conducted in this study.

Measurements were repeated if the Plusoptix device flagged low reliability (e.g., due to poor fixation, blinking, or small pupil size) or if fewer than three valid cycloplegic autorefraction readings could be obtained. The measurement was repeated until at least three reliable readings were available. Children without valid bilateral measurements after repeated attempts were excluded from the analysis.

Myopia was defined as SE ≤ −0.50 D in at least one eye, and high myopia as SE ≤ −6.00 D. For children with anisometropia (defined as an interocular difference in SE ≥ 1.00 D), each eye was analyzed separately, but classification of the participant as myopic was based on the more myopic eye.

### 2.4. Statistical Analysis

Statistical analysis was conducted using R (version 4.3.1) software. The normal distribution of variables was confirmed with the Kolmogorov–Smirnov test. The level of significance was established at 0.05 and prevalence calculated with a 95% confidence interval. The prevalence of myopia was calculated as the number of children with confirmed myopia after cycloplegic refraction, divided by the total number of children examined in each age group. Analyses comparing qualitative variables were resolved using the chi-square test and a univariate and multivariate logistic regression model was used to obtain the odds ratios (ORs). Variables with *p* < 0.10 in univariate analyses or considered clinically relevant according to prior literature were entered into the multivariate logistic regression model. Potential multicollinearity among independent variables (e.g., screen time, near work, and indoor activities) was assessed using the variance inflation factor (VIF) and tolerance values; no collinearity was detected (all VIF < 2). Model fit was evaluated using the Nagelkerke R^2^ and the Hosmer–Lemeshow goodness-of-fit test.

## 3. Results

Of the 3680 children enrolled in the schools selected, 2616 parents (71%) gave permission for their children to participate in the study and of these, 2489 (67%) were examined. 50.9% of the children were male, 1183 (47.2%) were in second grade (6–7 years) and 1306 (52.8%) in sixth grade (11–12 years).

The prevalence of myopia was 6.5% in second grade children [5.25–7.87], and 18.7% in sixth grade children [CI: 16.56–20.93] (*p* < 0.001). Mean myopia refraction was −1.98 (SD 1.76) in the second grade and −2.12 (SD 1.76) in sixth grade (*p* < 0.001). At least 32.3% of the children had a family history of myopia in one parent and 33.8% in both parents, 4.2% had a family history of high myopia in one or both parents.

The results of the analysis of associated factors for myopia are shown in Table 1 and more detailed in Table S1. The multivariate regression analysis confirmed that a family history of myopia in either parent was the only associated factor with the occurrence of myopia in children in 2nd grade. The presence of myopia in children in 6th grade was associated with family history of myopia in father and spending 2–6 h and more than six hours during the weekend in outdoor activities were factors associated with lower odds of myopia confirmed by the multivariate regression analysis.

The odds ratio for myopia in children if the mother had myopia was 1.99 (1.52–2.60, *p* < 0.001) and if father had myopia was 1.93 (1.47–2.54, *p* < 0.001) in the univariate analysis. If both parents had myopia the odds ratio for myopia in children if both parents had myopia was 2.04 (1.53–2.70, *p* < 0.001) in the univariate analysis. In the multivariate model (Table 1), only the combined parental myopia variable was included, as including separate maternal and paternal variables would have introduced collinearity.

We studied children’s habits on weekdays and weekends. On weekdays, most children spent 2–6 h per week outdoors, with smaller groups spending less than 2 h or more than 6 h. On weekends, outdoor time increased, with the majority spending 2–6 h and only a minority spending less than 2 h. Children with myopia were more frequently observed in the lower outdoor activity categories compared to non-myopic peers (*p* < 0.001).

Regarding screen time, most children reported 2–6 h both on weekdays and weekends, although a substantial proportion spent less than 2 h. On weekends, the proportion spending >6 h was slightly higher. Children with myopia were more frequently observed in the higher screen-time categories compared with non-myopic children (*p* < 0.001). In Tables S2 and S3 the habits of Madrid primary school students are described in more detail.

## 4. Discussion

As in the rest of the world, in Spain there is concern among families and ophthalmologists that the prevalence of myopia might be showing an increasing trend. According to the results of our study, the prevalence of myopia was significantly higher in children in sixth grade (11–12 years) compared to those in second grade (6–7 years) (18.7% vs. 6.5%). This difference likely reflects grade-specific environmental exposures—such as increased academic demands and reduced time outdoors—rather than age alone. These findings are consistent with previous studies showing that the prevalence of myopia increases with the number of years spent in school [13]. These results are comparable to those reported in other European countries. For example, Czepita et al. [14] in Poland observed a prevalence of myopia of 2.4% at 6 years, 8.4% at 8 years and 14.7% at 12 years, using cycloplegic refraction. The Irish Eye Study results, that also used cycloplegic refraction, showed a prevalence of 3.3% in children aged 6–7, slightly lower than our results; and 19.9% in children aged 12–13 [15], and an investigation in Germany found myopia in 11.6% in children aged 0–17 [16]. On the other hand, the prevalence of myopia in Spain is far from that in China, where Zhang et al. reported a prevalence of 60% among primary school students in Shenyang [9]. The lower prevalence of myopia in Spanish children compared with East Asian populations may be partly explained by environmental and lifestyle factors. Spain’s southern latitude and mild climate encourage outdoor play and greater daylight exposure, while educational schedules tend to be less intensive in early childhood than in East Asia. These conditions likely contribute to a lower cumulative visual strain and help protect against myopia development.

A detailed comparison with the study by Álvarez-Peregrina et al. [7] further highlights the importance of methodology when interpreting prevalence figures. Their study, which reported a 20.1% prevalence of myopia in Spanish children aged 5 to 7, used non-cycloplegic refraction and recruited participants through a voluntary campaign in optometric centers. This approach likely introduced selection bias, as families concerned about their children’s vision may have been more motivated to participate. In contrast, our study employed cycloplegic refraction and a school-based sampling strategy, yielding a prevalence of 6.5% among second grade children (6–7 years old). Given that cycloplegia is known to reduce false positives due to accommodation, and that school-based sampling enhances representativeness, these methodological differences likely explain the substantial disparity in reported prevalence. These findings underscore the need for standardized protocols in epidemiological studies of myopia to ensure comparability and accuracy.

In Europe the most current meta-analysis by Moreira-Rosário et al. [8] specifically stratified prevalence estimates by refraction method, reporting a myopia prevalence of 18.9% (95% CI: 13.2–26.5%) in cycloplegic studies versus 31.2% (95% CI: 24.9–38.3%) in non-cycloplegic studies. This demonstrates that non-cycloplegic methods substantially overestimate myopia prevalence, especially in children and adolescents, due to accommodation effects. The European Eye Epidemiology (E(3)) Consortium meta-analyses, used non-cycloplegic refraction and reported higher prevalence rates in adults (30.6% for myopia) [17].

Dragomirova et al. [18] reported a 14.2% prevalence of myopia in Bulgarian children in age group 6–10 and 19.9% in age group 11–15, with a non-cycloplegic refraction. These results seem to be higher than the myopia prevalence and enhance the importance of cycloplegic refraction when measuring myopia in children. Myopia prevalence in different countries is compared in Table 2.

Recent global projections by the Brien Holden Vision Institute estimate that by 2050, approximately 50% of the world’s population and 56% of the European population will be myopic [1]. These forecasts are primarily driven by data from East Asia, where myopia prevalence in school-aged children already exceeds 50%. The IMI (International Myopia Institute) white papers project dramatic increases in myopia prevalence, but these estimates are based on aggregated data from studies using both cycloplegic and non-cycloplegic methods and thus may overstate true prevalence in younger populations [24]. In contrast, our study, which used cycloplegic refraction in a representative school-based sample, found a prevalence of only 18.7% in Grade 6 children (aged 11–12 years) in Madrid. Given that children in Spain complete 12 years of schooling, as in most European countries, it is difficult to envision a threefold increase in myopia prevalence over the next 25 years without major shifts in educational or lifestyle patterns. Our results highlight the importance of regional data and the need to interpret global forecasts with caution.

As with many other chronic diseases, the development of myopia is related to genetic and environmental factors. Regarding family history, according to our results the risk of myopia is 50% higher if one or both parents have myopia. This risk is higher than that previously reported in Alvarez-Peregrina with an OR of 1.28 [7]. Our results are more similar to those published by Zhang et al. where they found an OR of 2.16 if the father is myopic, 2.00 if the mother is myopic and 2.94 if both parents are myopic [9]. As in previous studies, our results show that the most important factor related to the development of myopia is parental myopia [25]. Nonetheless, in our study, as in the others, parental data are self-reported and myopia in parents was not confirmed by ophthalmological examination, which is a limitation when interpreting these results.

While parental myopia is commonly interpreted as a genetic risk factor, it is increasingly recognized that this association may also reflect environmental influences. Myopic parents may be more likely to create conditions that promote myopia in their children, such as encouraging near work or limiting outdoor activities. Recent evidence from Guggenheim et al. [26] supports the idea that a substantial part of the intergenerational association is mediated by shared environmental exposures rather than heredity alone.

Regarding the influence of sex and myopia, although the results are not entirely consistent, the prevalence of myopia is higher in females than males, as reported previously in the literature [9,22]. Our results found a higher prevalence of myopia in girls but there were no statistical differences. He et al. proposed that sex differences in myopia could be due to girls being less active outdoors and spending more time reading and writing compared to boys [27]. This seems to be a reasonable explanation since the education of girls have changed significantly over the last 50–100 years. In turn, Xie et al. [28] suggested that myopia could be linked to hormonal changes during puberty, as high estrogen levels in adolescence could alter the shape of the eye, leading to myopia. However, Enthoven et al. [29] showed that gender differences in myopia are dynamic and likely reflect broader social and educational changes. In their Dutch cohort, boys had higher prevalence of myopia in earlier generations, whereas girls now show slightly higher prevalence in younger cohorts —possibly due to increased educational engagement and differences in lifestyle, such as lower outdoor activity. Insofar as family history, sex and genetic factors will remain unchanged over time, these results imply that we should pay close attention to environmental factors and habits affecting the development of myopia.

Children with myopia tended to spend more time in front of screens compared to those without myopia (*p* < 0.001). These results suggest that less exposure to outdoor activities and more screen time are associated with a higher prevalence of myopia in children. However, Screen time showed no independent association with myopia after adjusting for near-work and outdoor activity. The absence of an independent association in the multivariate model likely reflects overlap with other correlated behaviors, such as near work and reduced outdoor activity. From a public health perspective, excessive screen use may still contribute indirectly to myopia risk by displacing time outdoors and increasing sustained near-focus demands. Therefore, recommendations promoting balanced screen use and adequate outdoor time remain strongly supported.

On the other hand, more time spent on outdoor activities was found to be associated with lower prevalence of myopia in older children. It has been proposed that the protective effect of being outdoors against myopia involves the photostimulation of dopamine release in the retina: increased dopamine release seems to inhibit the axial elongation, which is the structural basis of myopia [30]. Schools and parents should be advised to increase time outdoors since it is the main protective factor against the development of myopia. In the questionnaire responses parents reported that 31.7% of children spent less than two hours a week outdoors on weekdays. These data may be due to the fact that Spanish children’s habits are changing, although it is more likely due to the fact that the study was conducted in 2021 when some families preferred to stay at home due to COVID-19.

Over the past decade, electronic devices such as smartphones, tablets and computers have increasingly dominated children’s leisure and entertainment time, and children are being exposed to screens at an increasingly younger age, even before the age of 2. Therefore, whether screen time, as a potentially modifiable risk factor, increases the prevalence of myopia has become an urgent concern for parents and ophthalmologists. Currently, the results regarding screen use and increased risk of myopia are inconclusive: while some studies find screens to be a possible risk factor [31], others found no direct effect between myopia and screen time [10]. In our study, although children with myopia were more frequently observed in higher screen-time categories, this association did not remain significant in the multivariate model. A possible explanation is collinearity with other lifestyle factors, particularly near work and time outdoors, which are conceptually and behaviorally related to screen use. This overlap may have masked an independent contribution of screen time. Similar difficulties in disentangling screen time from other near work exposures have been reported in previous epidemiological studies, highlighting the need for more refined and standardized measures of digital device use. Since screen time seems to be increasing in Spain with more schools using it for educational purposes, these findings should be revisited over time, but we did not find any evidence to support the limitation of screens at school and the return to book-reading.

Although socioeconomic status was considered using the Human Development Index (HDI) of the school districts, individual-level data—such as parental income, education, or occupation—were not collected. This limits the ability to analyze the specific influence of socioeconomic conditions on myopia development. Future studies should incorporate individual-level socioeconomic data to better understand its interaction with environmental and behavioral risk factors for myopia.

Although there are many epidemiologic studies worldwide determining the prevalence of myopia, few studies have been conducted in Spain and our work is the largest using cycloplegic refraction to determine the presence of myopia. However, despite the large sample size, as this is a cross-sectional study we are unable to reach conclusions regarding the causality of myopia. A cohort study should be performed to confirm our findings.

Our study exhibits several notable strengths that enhance the reliability of its findings. The use of cycloplegic refraction minimizes measurement errors related to accommodation, providing accurate assessments of myopia. Additionally, the large, representative sample of 2489 children from 39 randomly selected schools in Madrid ensures a robust dataset and enhances the generalizability of the results. We also conducted a comprehensive evaluation of both genetic factors, such as parental myopia, and environmental factors, including outdoor activity and screen time, with potential confounders controlled through multivariate logistic regression. Nonetheless, the study has certain limitations. Its cross-sectional design precludes causal inferences, and reliance on self-reported data for parental history and lifestyle factors may introduce recall bias. Furthermore, while socioeconomic status was accounted for using the Human Development Index of school districts, individual-level socioeconomic status data were not collected. Finally, the findings, while relevant to urban Madrid, may not be fully generalizable to rural areas or regions with different socio-demographic profiles.

A major limitation of this study is the use of a non-validated parental questionnaire to assess lifestyle and family history. Although the questionnaire was designed by a panel of experts, it was not pilot-tested or validated, which may have introduced recall and classification bias. This approach relies entirely on parental recall, raising concerns about reliability, recall bias, and comparability with other epidemiological studies. In addition, the questionnaire categories may conceptually overlap; for example, one hour spent on a computer could be classified simultaneously as indoor activity, screen time, and near work, leading to possible double counting and misclassification. Key variables, such as viewing distance during near work and continuity of activities, were not captured, nor was a distinction made between reading printed books and digital device use. Parents completed the questionnaire independently at home after receiving written instructions, but no pilot testing was performed. Furthermore, the instrument was not aligned with international standards. Future research in Spain should incorporate validated questionnaires and harmonized cycloplegia protocols to enhance reproducibility and comparability across populations.

Another important limitation of our study is the relatively low participation rate, as only 67% of the eligible children underwent examination. This introduces the possibility of selection bias, as non-participating children may differ systematically from those who took part. If, for example, children with no visual complaints or those from lower socio-economic backgrounds were less likely to participate, our prevalence estimates may be inflated. In an extreme scenario where none of the non-participating children were myopic, the actual prevalence could be significantly lower than reported. Future studies should aim to enhance participation through improved engagement strategies, such as additional parental outreach, flexible scheduling, and school-based incentives.

In conclusion, this cross-sectional study of 2nd and 6th grade schoolchildren in Madrid, we determined a myopia prevalence of 6.5% in second graders and 18.7% in sixth graders. The prevalence of myopia in primary school students in Madrid is comparable to that of other European countries, and, although these values remain far below those reported in East Asia, this should be carefully monitored. Our findings indicate that older age and a family history of myopia are significant risk factors, while increased outdoor activity appears to be associated with lower myopia prevalence. By employing cycloplegic refraction, our study provides more accurate estimates of myopia prevalence compared to previous reports using non-cycloplegic methods. These results highlight the importance of early detection and the promotion of lifestyle interventions aimed at increasing outdoor exposure as potential strategies for myopia prevention. Limitations include reliance on a non-validated questionnaire, potential instability of estimates in small subgroups, and limited generalizability beyond the study population. In addition, some associations observed in univariate analyses, such as screen time, were not confirmed in multivariate models. Future longitudinal studies are warranted to further elucidate the progression of myopia and the interplay between genetic and environmental factors in this population.

## Figures and Tables

**Table 1:** Analysis of associated factors for myopia primary school students in Madrid.

				2nd Grade	6th Grade
Variable	Levels	With Myopia N (%)	Without Myopia N (%)	OR (CI95%, *p*) Multivariate Analysis	OR (CI95%, *p*) Multivariate Analysis
Gender	Male	1136 (52.0)	132 (43.3)	-	-
	Female	1048 (48.0)	173 (56.7)	-	1.27 (0.76–2.11, 0.35)
Socio-economic status	Upper	182 (8.3)	31 (10.2)	-	-
	Upper middle	834 (38.2)	112 (36.7)	0.67 (0.26–1.70, 0.40)	-
	Middle	802 (36.7)	90 (29.5)	0.74 (0.29–1.87, 0.52)	-
	Lower	366 (16.8)	72 (23.6)	1.62 (0.61–4.21, 0.33)	-
Family history of myopia	Myopia Father	581 (26.6)	111 (37.0)	5.09 (1.49–17.30, 0.01)	7.85 (2.52–24.37, <0.01)
	High Myopia Father (<−6D)	45 (2.1)	19 (6.2)	-	-
	Myopia Mother	705 (32.3)	133 (44.4)	2.63 (1.19–5.78, 0.02)	2.58 (0.76–9.08, 0.14)
	High Myopia Mother (<−6D)	59 (2.7)	23 (7.5)	-	-
	Myopia Both	705 (32.3)	133 (43.6)	-	-
	High Myopia Both (<−6D)	20 (0.9)	12 (3.9)	-	-
Hours in different activities Monday to Friday					
Number of hours spent on outdoor activities	Less than 2 h	687 (31.5)	101 (33.1)	-	-
	Between 2 h and 6 h	943 (43.2)	111 (36.4)	-	-
	More than 6 h	269 (12.3)	26 (8.5)	-	5.16 (0.94–28.20, 0.06)
	No Answer	144 (6.6)	39 (12.8)	-	-
Number of hours spent on indoor activities that do not require excessive near vision	Less than 2 h	708 (32.4)	96 (31.5)	0.37 (0.11–1.15, 0.09)	-
	Between 2 h and 6 h	605 (27.7)	80 (26.2)	0.75 (0.25–2.22, 0.61)	-
	More than 6 h	47 (2.2)	6 (2.0)	0.45 (0.09–2.12, 0.31)	-
	No Answer	219 (10.0)	46 (5.1)	-	-
Number of hours spent on indoor activities that require near vision	Less than 2 h	672 (30.8)	74 (24.3)	0.37 (0.11–1.15, 0.09)	-
	Between 2 h and 6 h	877 (40.2)	123 (40.3)	0.75 (0.25–2.22, 0.61)	-
	More than 6 h	225 (10.3)	41 (13.4)	0.45 (0.09–2.12, 0.31)	-
	NA	167 (7.6)	35 (11.5)	-	-
Number of hours using electronic devices at near distance	Less than 2 h	813 (37.2)	97 (31.8)	-	-
	Between 2 h and 6 h	537 (24.6)	90 (29.5)	-	-
	More than 6 h	234 (10.7)	40 (13.1)	-	-
	No Answer	402 (18.4)	63 (20.7)	-	-
Hours in different activities on weekends (Saturday and Sunday)					
Number of hours spent on outdoor activities	Less than 2 h	356 (16.3)	75 (24.6)	-	-
	Between 2 h and 6 h	1384 (63.4)	150 (49.2)	-	0.18 (0.05–0.65, 0.04)
	More than 6 h	274 (12.5)	26 (8.5)	-	0.17 (0.04–0.73, 0.02)
	No Answer	124 (5.7)	32 (10.5)	-	
Number of hours spent on indoor activities that do not require excessive near vision	Less than 2 h	735 (33.7)	99 (32.5)	-	0.41 (0.16–1.00, 0.05)
	Between 2 h and 6 h	423 (19.4)	46 (15.1)	-	0.56(0.21–1.41, 0.22)
	More than 6 h	33 (1.5)	3 (1.0)	-	0.37 (0.04–3.32, 0.37)
	No Answer	254 (11.6)	52 (17.0)	-	-
Number of hours spent on indoor activities that require near vision	Less than 2 h	717 (32.8)	82 (26.9)	-	-
	Between 2 h and 6 h	900 (41.2)	131 (43.0)	-	-
	More than 6 h	52 (2.4)	5 (1.6)	-	-
	No Answer	188 (8.6)	42 (13.8)	-	-
Number of hours using electronic devices at near distance	Less than 2 h	542 (24.8)	53 (17.4)		
	Between 2 h and 6 h	1244 (57.0)	177 (58.0)		
	More than 6 h	208 (9.5)	44 (14.4)		
	No Answer	114 (5.2)	26 (8.5)		
Screen time					
Before bedtime screen use	Yes	835 (38.2)	137 (44.9)	6.27 (0.28–Inf, 0.25)	
	No	1271 (58.2)	149 (48.9)	5.86 (0.20–Inf, 0.24)	
Early morning screen use	Yes	309 (14.1)	57 (18.7)	0.17 (0.00–4.17, 0.30)	
	No	1798 (82.3)	229 (75.1)	0.16 (0.00–3.62, 0.25)	

**Table 2:** Prevalence of myopia in different countries.

Country	Year Published	Number of Patients	Refraction Technique	Age of Patients	Myopia Prevalence
Ethiopia [19]	2023	20,757	C and NC	School-aged children	5.26%
Multiple countries [3]	2022	36,395	C and NC	5–11 years	3.4%
India [20]	2017	9616	C	5–15 year	3.4%
India [6]	2022	13,572	C	6–15 years	3.7%
China [9]	2023	34,644	NC	11.9 years	60%
China [5]	2022	721,032	NC	11.53 years	51.8%
China [21]	2025	218,794	C	5–9 years	22%
				10–14 year	45.4%
Bulgaria [18]	2022	1401	NC	6–10 years	14.2%
				11–15 years	19.9%
Spain [13]	2021	1601	NC	5–7 years	19.7%
Poland [22]	2019	4875	C	6–13	8.30% boys 5.71% girls
Ireland [15]	2020	1626	C	6–7 year	3.3%
				12–13 year	19.9%
Germany [16]	2020	15,023	NC	0–17 year	11.4%
Netherlands [23]	2023	6032	C	6 years	2.4%
				13 years	22.5%

Legend: C—Cycloplegic; NC—Non-cycloplegic.

## Data Availability

Data is unavailable due to privacy.

## Supplementary Materials

The following supporting information can be downloaded at: https://www.mdpi.com/article/10.3390/ijerph22121766/s1; File S1. Parents questionnaire on child’s habits (what activities he/she does after school); Table S1: Analysis of associated factors for myopia primary school students in Madrid; Table S2. Habits of Madrid primary school students Monday to Friday; Table S3. Habits of Madrid primary school students during weekends.
