# Supplementary material for: Prevalence and Risk Factors for Myopia in Primary School Children in Madrid: A School-Based Cycloplegic Refraction Study
Source: Int J Environ Res Public Health. 2025 Nov 21;22(12):1766. doi: 10.3390/ijerph22121766 (PMC12733093; doi:10.3390/ijerph22121766)
Supplement: Supplementary file 1 [file ijerph-22-01766-s001.zip › ijerph-3943228-supplementary.pdf]

File S1. Parent questionnaire on child's habits (what activities **they do** after school).

Type of activity	Total hours during the week (Monday to Friday)					Total hours weekends (Saturday and Sunday)				
	Less than 2h	2-4h	4-6h	6-8h	More than 8h	Less than 2h	2-4h	4-6h	6-8h	More than 8h
**<u>Outdoor</u> activity (e.g. soccer, athletics, playing in the park, etc.)**										
**<u>Indoor</u> activities that do not require excessive near vision (e.g. basketball, swimming, paddle tennis, gymnastics, dance, etc.).**										
**Indoor activities with importance of near vision work (e.g. painting, language study, reinforcement, etc.).**										
**Activity with electronic devices at a distance of less than 50 cm (e.g. cell phones, tablet, videogames, etc.).**										

(Mark with a cross where appropriate) (2h =2 hours, 4h= 4 hours)

Myopia and electronic devices

It is being studied whether there is a relationship between myopia and the use of electronic devices.

- Does the child perform activities with electronic devices (cell phones, Tablet, consoles, etc.) during the last hour before going to bed? □YES□ NO
- Does the child perform activities with electronic devices (cell phones, Tablet, consoles, etc.) first thing in the morning? □YES□ NO

Table S1. Analysis of associated factors for myopia primary school students in Madrid.

				2nd Grade		6th Grade	
**Variable**	**Levels**	**With myopia N (%)**	**Without myopia N (%)**	**OR (CI95%, p) Univariate analysis**	**OR (CI95%, p) Multivariate analysis**	**OR (CI95%, p) Univariate analysis**	**OR (CI95%, p) Multivariate analysis**
Gender	Male	1136 (52.0)	132 (43.3)				
	Female	1048 (48.0)	173 (56.7)	1.19 (0.72-1.93, 0.49)		1.43 (1.07-1.90, **0.014**)	1.27 (0.76-2.11, 0.35)
Socio-economic status	Upper	182 (8.3)	31 (10.2)				
	Upper middle	834 (38.2)	112 (36.7)	0.59 (0.33-1.00, 0.59)	0.67 (0.26-1.70, 0.40)	1.09 (0.81-1.45, 0.54)	
	Middle	802 (36.7)	90 (29.5)	0.80 (0.46-1.33, 0.41)	0.74 (0.29-1.87, 0.52)	0.67 (0.49-0.90, **0.01**)	
	Lower	366 (16.8)	72 (23.6)	2.31 (1.33-2.29, **0.002**)	1.62 (0.61-4.21, 0.33)	1.35 (0.94-1.90, 0.10)	
Family history of myopia	Myopia Father	581 (26.6)	111 (37.0)	1.39 (0.82-2.29, 0.21)	5.09 (1.49-17.30, **0.01**)	1.61 (1.20-2.14, **<0.01**)	7.85 (2.52-24.37, **<0.01**)
	High Myopia Father (<-6D)	45 (2.1)	19 (6.2)	6.15 (2.15-15.36, **0.018**)		2.17 (1.07-4.17, **0.02**)	
	Myopia Mother	705 (32.3)	133 (44.4)	1.93 (1.18-3.14, **0.008**)	2.63 (1.19-5.78, **0.016**)	1.61 (1.20-2.14, **<0.01**)	2.58 (0.76-9.08, 0.14)
	High Myopia Mother (<-6D)	59 (2.7)	23 (7.5)	1.95 (0.57-5.09, 0.218)		3.66 (1.95-6.74, **<0.01**)	
	Myopia Both	705 (32.3)	133 (43.6)	1.93 (1.18-3.14, **0.008**)		1.61 (1.20-2.14, **<0.01**)	
	High Myopia Both (<-6D)	20 (0.9)	12 (3.9)	7.83 (2.40-22.25, **<0.001**)		3.60 (1.27-9.77, **0.01**)	
**Hours in different activities Monday to Friday**							
Number of hours spent on outdoor activities	Less than 2h	687 (31.5)	101 (33.1)	0.94 (0.54-1.56, 0.80)		1.14 (0.84-1.53, 0.39)	
	Between 2h and 6h	943 (43.2)	111 (36.4)	1.10 (0.67-1.78, 0.71)		0.69 (0.50-0.92, **0.01**)	
	More than 6h	269 (12.3)	26 (8.5)	0.60 (0.22-1.30, 0.25)		0.75 (.044-1.21, 0.27)	5.16 (0.94-28.20, 0.06)
	NA	144 (6.6)	39 (12.8)				
	Less than 2h	708 (32.4)	96 (31.5)	1.10 (0.64-1.18, 0.16)	0.37 (0.11-1.15, 0.09)	0.93 (0.67-1.25, 0.65)	

Number of hours spent on indoor activities that do not require excessive near vision	Between 2h and 6h	605 (27.7)	80 (26.2)	1.29 (0.76-2.12, 0.32)	0.75 (0.25-2.22, 0.61)	0.93 (0.66-1.25, 0.62)	
	More than 6h	47 (2.2)	6 (2.0)	0.00 (0.00, 0.98)	0.45 (0.09-2.12, 0.31)	0.94 (0.34-2.13, 0.59)	
	NA	219 (10.0)	46 (5.1)				
Number of hours spent on indoor activities that require near vision	Less than 2h	672 (30.8)	74 (24.3)	0.53 (0.28-0.91, **0.03**)	0.37 (0.11-1.15, 0.09)	0.98 (0.70-1.35, 0.90)	
	Between 2h and 6h	877 (40.2)	123 (40.3)	1.67 (1.02-2.71, **0.04**)	0.75 (0.25-2.22, 0.61)	0.78 (0.58-1.04, 0.10)	
	More than 6h	225 (10.3)	41 (13.4)	0.63 (0.15-1.74, 0.44)	0.45 (0.09-2.12, 0.31)	1.18 (0.78-1.71, 0.41)	
	NA	167 (7.6)	35 (11.5)				
Number of hours using electronic devices at near distance	Less than 2h	813 (37.2)	97 (31.8)	0.93 (0.56-1.15, 0.76)		0.93 (0.67-1.16, 0.66)	
	Between 2h and 6h	537 (24.6)	90 (29.5)	0.75 (0.36-1.38, 0.38)		1.23 (0.90-1.65, 0.18)	
	More than 6h	234 (10.7)	40 (13.1)	1.77 (0.59-4.22, 0.24)		0.82 (0.54-1.20, 0.32)	
	NA	402 (18.4)	63 (20.7)				
**Hours in different activities on weekends (Saturday and Sunday)**							
Number of hours spent on outdoor activities	Less than 2h	356 (16.3)	75 (24.6)	0.94 (0.42-1.83, 0.86)		1.67 (1.20-2.29, **<0.01**)	
	Between 2h and 6h	1384 (63.4)	150 (49.2)	0.83 (0.50-1.38, 0.47)		0.50 (0.37-0.66, **<0.01**)	0.18 (0.05-0.65, **0.04**)
	More than 6h	274 (12.5)	26 (8.5)	0.82 (0.37-1.59, 0.57)		0.82 (0.54-1.20, 0.33)	0.17 (0.04-0.73, **0.02**)
	NA	124 (5.7)	32 (10.5)				
Number of hours spent on indoor activities that do not require excessive near vision	Less than 2h	735 (33.7)	99 (32.5)	0.85 (0.48-1.41, 0.53)		0.99 (0.73-1.33, 0.97)	0.41 (0.16-1.00, 0.05)
	Between 2h and 6h	423 (19.4)	46 (15.1)	1.20 (0.64-2.11, 0.54)		0.60 (0.39-0.89, **0.02**)	0.56(0.21-1.41, 0.22)
	More than 6h	33 (1.5)	3 (1.0)	0.85 (0.04-4.17, 0.87)		0.64 (0.10-2.32, 0.56)	0.37 (0.04-3.32, 0.37)
	NA	254 (11.6)	52 (17.0)				
Number of hours spent on indoor activities	Less than 2h	717 (32.8)	82 (26.9)	0.75 (0.43-1.25, 0.29)		0.87 (0.62-1.18, 0.38)	
	Between 2h and 6h	900 (41.2)	131 (43.0)	1.02 (0.6-1.67, 0.9)		0.97 (0.72-1.28, 0.82)	

that require near vision	More than 6h	52 (2.4)	5 (1.6)	0.00 (0.00, 0.98)		0.60 (0.20-1.42, 0.30)	
	NA	188 (8.6)	42 (13.8)				
Number of hours using electronic devices at near distance	Less than 2h	542 (24.8)	53 (17.4)	0.91 (0.52-1.51, 0.71)		0.78 (0.51-1.15, 0.23)	
	Between 2h and 6h	1244 (57.0)	177 (58.0)	0.91 (0.55-1.47, 0.70)		0.93 (0.69-1.23, 0.60)	
	More than 6h	208 (9.5)	44 (14.4)	1.03 (0.24-2.91, 0.96)		1.19 (0.80-1.71, 0.37)	
	NA	114 (5.2)	26 (8.5)				
**Screen time**							
Before bedtime screen use	Yes	835 (38.2)	137 (44.9)	2.22 (1.35-3.61, **0.001**)	6.27 (0.28-Inf, 0.25)	0.87 (0.65-1.15, 0,33)	
	No	1271 (58.2)	149 (48.9)	0.38 (2.23-0.62, 0.00)	5.86 (0.20-Inf, 0.24)	1.05 (0.79-1.39, 0.73)	
Early morning screen use	Yes	309 (14.1)	57 (18.7)	1.56 (0.76-3.01, 0.21)	0.17 (0.00-4.17, 0.30)	1.08 (0.75-1.53, 0.64)	
	No	1798 (82.3)	229 (75.1)	0.49 (0.27-0.88, **0.014**)	0.16 (0.00-3.62, 0.25)	0.84 (0.6-1.16, 0.28)	

Table S2. Habits of Madrid primary school students Monday to Friday.

**Hours in different activities Monday to Friday**		**2nd Grade**		**6th Grade**	
		**With myopia %**	**Without myopia %**	**With myopia %**	**Without myopia %**
Number of hours spent on outdoor activities	Less than 2h	37.2	35.9	40.9	45.5
	Between 2h and 6h	43.7	46.6	41.7	32.7
	More than 6h	12.7	7.3	10.4	7.9
	NA	6.4	10.3	6.9	13.9
Number of hours spent on indoor activities that do not require excessive near vision	Less than 2h	57.7	48.2	62.7	61.9
	Between 2h and 6h	30.0	35.2	24.1	20.6
	More than 6h	2.0	0.0	2.6	2.1
	NA	10.2	16.6	10.7	15.4
Number of hours spent on indoor activities that require near vision	Less than 2h	47.4	32.7	36.4	38.6
	Between 2h and 6h	37.6	44.9	42.5	36.1
	More than 6h	6.4	3.1	14.0	15.4
	NA	8.7	19.3	7.1	9.9
Number of hours using electronic devices at near distance	Less than 2h	56.0	45.7	34.6	31.4
	Between 2h and 6h	21.4	18.9	29.5	34.6
	More than 6h	4.5	5.6	17.2	14.3
	NA	18.1	29.8	18.7	19.7

Table S3. Habits of Madrid primary school students during weekends.

Hours in different activities on weekends (Saturday and Sunday)		2nd Grade		6th Grade	
		With myopia %	Without myopia %	With myopia %	Without myopia %
Number of hours spent on outdoor activities	Less than 2h	15.9	13.5	23.9	37.7
	Between 2h and 6h	63.9	60.5	61.4	46.6
	More than 6h	14.2	10.6	9.2	5.7
	NA	6.0	15.4	5.6	10.0
Number of hours spent on indoor activities that do not require excessive near vision	Less than 2h	66.2	51.5	67.8	70.4
	Between 2h and 6h	19.2	21.3	19.2	13.4
	More than 6h	2.0	0.8	1.4	0.5
	NA	12.6	26.3	11.7	15.6
Number of hours spent on indoor activities that require near vision	Less than 2h	51.0	47.5	44.2	41.5
	Between 2h and 6h	37.6	35.4	44.3	44.4
	More than 6h	1.3	0.0	3.4	1.5
	NA	10.0	17.1	8.0	12.7
Number of hours using electronic devices at near distance	Less than 2h	36.8	33.6	18.1	14.8
	Between 2h and 6h	52.2	50.2	61.3	60.8
	More than 6h	4.6	3.3	16.2	16.7
	NA	6.4	12.9	4.4	7.6
